# Cerebral vein thrombosis in women using short course oral contraceptive consumption 

**Published:** 2012-11

**Authors:** Payam Sasannejad, Ali Mellat Ardekani, Arash Velayati, Ali Shoeibi, Morteza Saeidi, Mohsen Foroughipour, Mahmoud Reza Azarpazhooh

**Affiliations:** 1 *Department of Neurology, Ghaem Medical Center, Mashhad University of Medical Sciences, Mashhad, Iran. *; 2 *Department of Neurology, Shahid Sadoughi Hospital, Shahid Sadoughi University of Medical Sciences, Yazd, Iran.*

**Keywords:** *Cerebral vein thrombosis*, *Oral contraceptives*, *Menstruation*, *Ramadan*

## Abstract

**Background:** Muslim women tend to postpone their menstrual period using short course oral contraceptives (OC) during Ramadan, Muslims fasting month. Recently, many cases of stroke, particularly cerebral vein thrombosis (CVT), have been reported in Ramadan.

**Objective:** This work studies the incidence of CVT and its relation to Ramadan.

**Materials and Methods:** This is a cohort study conducted from October 2006 to October 2009 and included 4 consecutive Ramadan’s. All patients with definite diagnosis of CVT who were referred to the neurology department of Ghaem Hospital, Mashhad, Iran in this four-year period were included in the study.

**Results:** During the study period, 70 cases with CVT (11 males and 59 females) were recruited. Twenty five cases were admitted during Ramadan months which was significantly higher than all the other 32 months (p=1.9×10^-7^). The higher frequency of females (6 times more) among CVT cases probed by investigating possible risk factors and short term OC consumption was revealed as the major risk factor (p=0.00071).

**Conclusion:** Higher incidence of CVT in females during Ramadan suggests the presence of specific risk factors in this group. Our study revealed that short-term use of OCs may be a major risk factor for CVT.

## Introduction

The view on contraception and birth control varies among different countries, due to religion and cultural, socioeconomic and educational factors (1). Oral contraceptives (OC) consumption is one of the most widely used methods of birth control. 

Currently, more than 100 million women use OC worldwide (2). Despite the high levels of OC use in the United States, Europe, Latin America, and the Caribbean, the popularity of OC across Asian countries has remained relatively low (3). The use of contraception dates back to many years ago in Islamic countries. Rhazes and Avicenna, the most famous Persian scientists in the 9^th^ and 10^th^ century, listed many birth control substances in their books "The Canon of Medicine" and "Al-Hawi". Although, among Muslims, there is still some reluctance to use contraception, the fertility rate has decreased and contraceptive usage has increased in Islamic countries (4-9). Religious and cultural factors have also had a great influence on the pattern of OC usage. 

For instance, as Islamic law prohibits women from participating in some Islamic ceremonies during the menstrual period, Muslim women tend to use oral contraception as a tool to postpone menstruation. These prohibitions include entering the holy places in Mecca and Medina in Hajj ceremony or in Muslim mosques and also fasting in the Islamic month of Ramadan, the ninth and the holiest month of the Islamic calendar which is marked by praying and fasting. There are several reports about adverse vascular complications of short-term OC consumption during Hajj Ceremony and also a few studies about cerebral vein thrombosis (CVT) among Iranian Muslims during Ramadan (10). 

We have previously shown a higher incidence of CVT among OC consumers during Ramadan (11). However, to further investigate the relation between short-term OC consumption and CVT in Ramadan, this study presents the result of an additional investigation on a larger group of OC consumers.

## Materials and methods

In a cohort study, from October 2006 to October 2009, including 4 consequence Ramadans, all cases with diagnosis of CVT were registered and entered to the study. The study was conducted in the Neurology Department of Ghaem Hospital, Mashhad, Iran which is the main referral center of neurology in the Northeast of Iran. The description of case registry and evaluation is explained in details in our previous publication (11). 

In brief, CVT was diagnosed based on the clinical presentations and neuro imaging findings. Patients were evaluated for several CVT risk factors such as past or family history of venous thrombosis, hypercoagulopathy state, collagen vascular disorders, infectious disorders, complicated otitis, cancer, past history of trauma, drug abuse and history of migraine, as well as history of short and long-term consumption of OC and the type of OC based on the estradiol component.

**Statistical analysis**

The incidence of CVT during the Ramadan months in comparison with other months was analyzed by regression studies using SPSS version 11.5 (SPSS Inc, Chicago, IL, USA) statistical software. Chi-squared was used to investigate CVT relation to OC consumption. One-tailed *t*-test was applied to analyze demographic data with p-value less than 0.05 considered as statistically significant. This study was approved by the Ethic Committee of Mashhad University of Medical Sciences.

## Results

**Figure 1:**
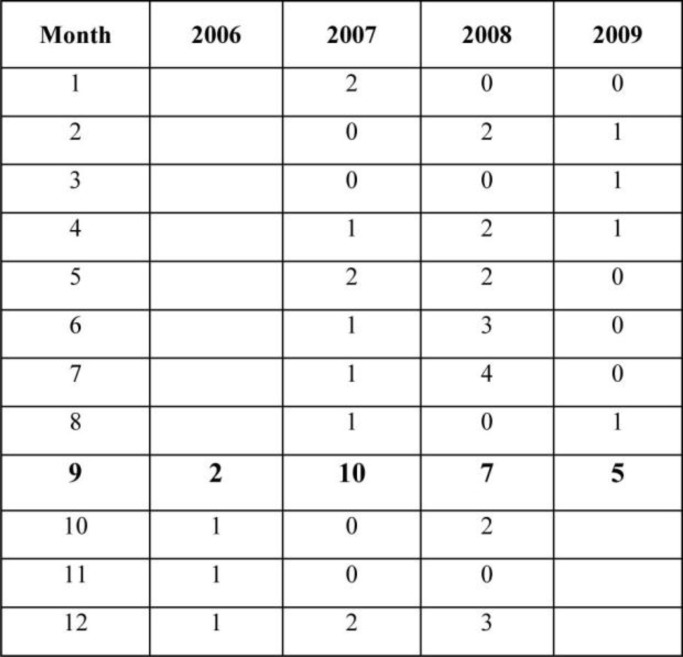
Distribution of CVT cases among females during study period. The order of the months is according to Arabic calendar, “Ramadan” is the ninth month of the calendar

During the study period, 70 cases (11 males and 59 females) with definite diagnosis of CVT entered the study. All patients had BMI below 29. Among these cases, 25 (24 females, 1 male) were admitted in Ramadan and 45 (35 females, 10 males) were admitted in months other than Ramadan. Regression analysis revealed that the incidence of CVT in Ramadan was 4.96 additional cases (95% CI: 3.37-6.43, p=0.000117) comparing to all the reminding 32 months which calculated to be 1.09 cases per month (95% CI: 0.58-1.60) (Figure 1). 

Statistical analysis of demographic data addressed OC consumption as the only risk factor with statistical significance (Table I). Excluding male patients from the analyses, 19 out of 24 female CVT cases in Ramadans had a history of short term OC consumption. In contrast, the OC consumption was found in 12 out of 35 female CVT cases discovered in non-Ramadan months (p=0.00071). Among the OC consumers in Ramadan, 17 cases used LD and 2 had a history of HD consumption. 

Surprisingly, no other risk factors were detected in this group. We also did not detect any risk factor in the 5 non OC consumers in this group. In contrast, among the CVT cases detected in non-Ramadan months, 12 cases consumed OC and 18 had other risk factors for CVT such as hyper coagulopathy state due to factor V Leiden (3 cases), systemic lupus erythematosus (2 cases), complicated otitis and mastoiditis (2 cases), pregnancy (4 cases), a history of recent (within 6 months) caesarian section (4 cases), and severe dehydration, history of oral corticosteroid use and esteradiol injection after severe uterine bleeding (each in 1 case) (Table I). 

The superior sagital sinus either with or without involvement of lateral sinuses was the most common site for CVT (Table II). Table II shows the clinical characteristics of women with CVT in Ramadan and non-Ramadan in which seizure as a symptom of CVT was observed more frequently in Ramadan group (p=0.006) as well as ischemic venous infarcts (p=0.017) (Table II). Overall, 6 patients died in the hospital and 6 were discharged with significant disability (Modified Rankin scale ≥3).

**Table I:** Demographic data of patients with cerebral vein thrombosis

**Demographics**		**Female** **(N= 59)**	**Male** **(N= 11)**
Age: Mean± standard deviation (years)		34 ± 7	36 ± 5
Risk factor			
	Short course OCP	23	-
	Long course OCP	8	-
	Hypercoaguability states	3	1
	Mastoiditis	2	4
	Pregnancy	4	-
	Cesarean section	4	-
	Systemic lupus erythematosus	2	-
	Other risk factors	1	3
	No apparent risk	12	3
Site of CVT			
	Superior sagital sinus	39	6
	Right lateral sinus	22	6
	Left lateral sinus	9	2

OCP: Oral contraceptive pill.

**Table II:** Clinical characteristics of women with CVT during study

**Characteristics**		**Ramadan** **(n=24)**	**Non Ramadan** **(n=35)**	**p-value**
Clinical features				
	Seizure	17	12	0.006
	Focal neurologic deficit	14	13	0.131
Imaging				
	Ischemic venous infarct	14	20	0.017
	Paranchymal hemorrhage	7	13	0.52
	Subarachnoid hemorrhage	5	5	0.50
Morbidity		3	3	1
Mortality		2	4	1

CVT: Cerebral vein thrombosis.

Chi-square test.

## Discussion

Oral contraceptives are associated with several major side effects including cardiovascular, cerebro vascular, and peripheral vascular disorders (12-16). Although several studies have investigated the safety and possible side effects of long-term OC consumption, short-term OC consumption requires more investigation (10). Venous thrombosis (both deep vein and cerebral vein thrombosis) are known side effects of OC consumption that might stem from a genetic susceptibility for hyper coagulopathic disorders (17-21). However, the safety and the real mechanism of vascular events, particularly in newer generation of OC, is still a matter of debate.

Our study aimed to investigate the relation between short-term OC consumption and CVT in Ramadan. This study clearly showed almost a five-fold increase in the incidence of CVT in Ramadan, in the presence of no other detectable risk factor other than OC consumption. Although we could not find any known predisposing factor in these patients, it seems that other predisposing factors in Ramadan such as fasting, dehydration, and stress may facilitate contraceptive side effects on vasculature and exacerbate thrombotic events associated with them. Presence of a genetic susceptibility factor might also be related to this high incidence of CVT in short term OC users.

We observed that CVT cases in Ramadan were in more severe conditions in terms of clinical presentations and outcome. As shown in table II, seizures and ischemic infarcts with neurologic sequels were more common in Ramadan group. This observation may have similar implications for practice. In order to reduce the mortality and morbidity of CVT among OC consumers during Ramadan preventive modalities need to be taken into consideration. Patients should be advised to have adequate hydration. They should also be monitored for any possible pre-thrombotic risk factors including infections, connective tissue diseases, malignancies and other known hyper coagulability states. Some studies reported that underlying factors could be identified in up to 80% of patients (22). 

In fact, the slow progression of thrombosis in the brain venous system gives the physician an opportunity to detect and treat the disease in early stages, when the morbidity can remain minimal. Physicians should be aware of early symptoms of CVT. Arguably, even a simple headache, for instance, in an OC consumer referring to ER or clinic in Ramadan can be a red flag of an evolving CVT.

## Conclusion

In conclusion, the short-term OC consumption may lead to serious vascular disorders such as CVT even in the absence of other known hyper coagulability conditions. This study suggests that the side effects of short term OC use may outweigh its benefits and thus, should be prescribed with utmost caution.
